# Conformational thermostabilisation of corticotropin releasing factor receptor 1

**DOI:** 10.1038/srep11954

**Published:** 2015-07-10

**Authors:** James Kean, Andrea Bortolato, Kaspar Hollenstein, Fiona H. Marshall, Ali Jazayeri

**Affiliations:** 1 Heptares Therapeutics Ltd, BioPark, Broadwater Road, Welwyn Garden City, AL7 3AX, UK

## Abstract

Recent technical advances have greatly facilitated G-protein coupled receptors crystallography as evidenced by the number of successful x-ray structures that have been reported recently. These technical advances include novel detergents, specialised crystallography techniques as well as protein engineering solutions such as fusions and conformational thermostabilisation. Using conformational thermostabilisation, it is possible to generate variants of GPCRs that exhibit significantly increased stability in detergent micelles whilst preferentially occupying a single conformation. In this paper we describe for the first time the application of this technique to a member of a class B GPCR, the corticotropin releasing factor receptor 1 (CRF_1_R). Mutational screening in the presence of the inverse agonist, CP-376395, resulted in the identification of a construct with twelve point mutations that exhibited significantly increased thermal stability in a range of detergents. We further describe the subsequent construct engineering steps that eventually yielded a crystallisation-ready construct which recently led to the solution of the first x-ray structure of a class B receptor. Finally, we have used molecular dynamic simulation to provide structural insight into CRF_1_R instability as well as the stabilising effects of the mutants, which may be extended to other class B receptors considering the high degree of structural conservation.

As one of the largest superfamilies of transmembrane receptors, G-protein coupled receptors (GPCRs) have long been targets for drug discovery due to their importance in signalling cascades throughout the body. This diverse family can recognise a wide variety of ligands ranging from small molecule neurotransmitters^1^,^2^ and metabolites^3^ to large peptide hormones^4^,^5^ and chemokines^6^. The complexity of GPCR function is compounded by their capacity to activate a multiplicity of downstream pathways, whether this be mediated through trimeric G-proteins (of which there are at least 17 different mammalian genes encoding G_α_ subtypes, 5 types of G_β_ and 12 types of G_γ_)^7^, arrestins^8^, or other mediators^9^. GPCRs can be divided into 4 major families according to sequence homology and functional similarity^10^. The majority of structural analysis has focussed on Class A receptors (rhodopsin-like) which make up the predominant class of GPCRs in the body^11^,^12^, however recent successes have provided structural examples from other families: Class B (secretin)^13^,^14^, Class C (metabotropic glutamate)^15^,^16^ and Class F (frizzled/smoothened)^17^.

The corticotropin releasing factor receptor 1 (CRF_1_R) is a member of the Class B, or secretin receptor family^4^,^18^. It is predominantly expressed in the pituitary and areas of the central nervous system, such as hypothalamus, amygdala and cortex, and plays a key role in the regulation of the hypothalamic pituitary adrenal stress axis in mammals^19^,^20^,^21^. Thus, antagonists of the receptor have been investigated as possible therapeutic agents to combat stress-related disorders like anxiety and depression^22^,^23^. Its natural ligand, corticotropin releasing factor (CRF), is a 41 amino acid peptide hormone that interacts with the receptor through both the 7 transmembrane helical bundle and a large amino-terminal extracellular domain (ECD); a characteristic of Class B GPCRs^24^. As with other members of its Class, the carboxy-terminus of the peptide ligand binds to the ECD of the receptor and the amino-terminus of the peptide interacts with the transmembrane domain (TMD). Structures of the ECD of CRF_1_R have been solved in complex with peptide ligands^25^,^26^; however, only recently the structure of the TMD of CRF_1_R was determined, bound with an inverse agonist, CP-376395^13^.

GPCRs are highly dynamic proteins and inherently unstable when removed from the lipidic environment of the membrane. This makes them challenging targets for structural studies. Stability and homogeneity are recognised traits that correlate with success in structural biology of membrane proteins^27^,^28^,^29^,^30^. These traits are in many ways linked. A level of homogeneity can be achieved through effective purification of the target protein, however monodispersity of the sample is in turn affected by its stability. This is especially relevant for membrane proteins where purification requires solubilisation in detergent micelles which are typically destabilising compared to the membrane^31^,^32^. Further heterogeneity can be present at a molecular level in the form of flexible regions such as loops, extended termini and mobile domains that can hinder crystallisation. These are typically removed or modified in the construct design of proteins that are difficult to crystallise^33^. Moreover, the dynamic nature of GPCRs and their ability to exist in a spectrum of conformations in the activation landscape creates additional diversity within the population^34^. We were able to overcome these difficulties and solve the structure of CRF_1_R by application of conformational thermostabilisation. This entailed the introduction of stabilising mutations throughout the receptor that locked the receptor into a single conformation, driven by the presence of the ligand. This stabilised receptor (StaR) generation process has been adopted successfully for a number of other GPCR targets, enabling high resolution structural analysis^11^,^15^,^35^,^36^,^37^.

In this paper we describe the generation of the CRF_1_R StaR through mutagenesis and the subsequent construct optimisation that enabled the receptor to be purified, crystallised, and its structure solved to 3 Å resolution. The thermal stability of the StaR has been characterised, highlighting the link between stability and crystallisability, and molecular dynamics simulation analyses point to a possible route of instability within the receptor as well as providing explanations for the stabilising effects of mutations.

## Methods

### StaR generation

The CRF_1_R StaR was generated using a mutagenesis approach described previously^36^. Mutants were analysed for thermostability in the presence of the radioligand [^3^H]CP-376395. Stabilising mutations were combined to create a CRF_1_R StaR containing 12 mutations.

### Cell culture

HEK293T cells were cultured in DMEM supplemented with 10% (v/v) fetal bovine serum (FBS). Cells were transfected using GeneJuice (Merck Millipore) according to manufacturer’s instructions and harvested after 48 hours using PBS supplemented with Complete EDTA-free protease inhibitors (Roche).

### Thermostability measurement

Transiently transfected HEK293T cells were incubated in 50 mM Tris-HCl pH 7.5, 150 mM NaCl, 2 × Complete EDTA-free protease inhibitors (PI) (Roche) with 30 nM [^3^H]CP-376395 and 120 nM cold CP-376395 (Tocris) for 18 hours at room temperature. All subsequent steps were performed at 4 °C. Cells were solubilized in either 1% (w/v) *n*-dodecyl-*β*-*d*-maltopyranoside (DDM) or 1% (w/v) *n*-decyl-*β*-*d*-maltopyranoside (DM) for 1 hour and crude lysates cleared by centrifugation at 16,000 × *g* for 15 minutes. Lysates were incubated with Ni-NTA resin (Qiagen), pre-equilibrated with sample buffer, for 1 hr 30 min before washing with 50 mM Tris-HCl pH 7.5, 150 mM NaCl, 20 mM Imidazole, supplemented with either 0.03% (w/v) DDM or 0.15% (w/v) DM. For detergent exchange experiments, DM solubilised samples were exchanged with wash buffer substituted with either 0.4% (w/v) *n*-nonyl-*β*-*d*-maltopyranoside (NM), 0.3% (w/v) *n*-nonyl-*β*-*d*-glucopyranoside (NG), 0.8% (w/v) *n*-octyl-*β*-*d*-glucopyranoside (OG), 0.4% (w/v) *n*-octyl-*β*-*d*-thioglucopyranoside (OTG), 0.35% (w/v) decanoyl-N-hydroxyethylglucamide (HEGA10), 0.35% (w/v) tetraethylene glycol monoctyl ether (C_8_E_4_), or 0.1% (w/v) n-dodecyl-n,n-dimethylamine-n-oxide (LDAO). Receptor was eluted from the resin using wash buffer containing 100 mM histidine, 30 nM [^3^H]CP-376395 and 120 nM cold CP-376395 and incubated at 4 °C for 1 hour. A_2A_R StaR samples were treated as above but using buffers containing 400 mM NaCl. These samples were eluted from the Ni-NTA resin using wash buffer containing 100 mM histidine and 100 nM [^3^H] ZM241385.

For buffer and additive screening experiments, the receptor was eluted from Ni-NTA resin using wash buffer containing 100 mM histidine. Histidine was removed and the buffer exchanged by passing the material through a PD-10 desalting column. Receptor was eluted in either 10 mM Tris-HCl, 150 mM NaCl, 2x PI, 0.15% (w/v) DM, pH 7.5 (for buffer exchange experiments) or 100 mM sodium citrate, 150 mM NaCl, 2x PI, 0.15% DM, pH 5.5 (for additive screen experiments), and incubated with 30 nM [^3^H]CP-376395 and 120 nM cold CP-376395 overnight. This material was then mixed in a 1:1 ratio with exchange buffer (200 mM buffer, 150 mM NaCl, 0.15% DM) or additive buffer (100 mM sodium citrate, 150 mM NaCl, 0.15% DM, 400 mM additive, pH5.5) and incubated at 4 °C for 5 min. Buffers were chosen based upon optimum buffering capacity at the given pH.

Thermostability of the receptor was measured by incubation at varying temperatures for 30 minutes followed by separation of unbound radioligand by gel filtration as described previously^38^. Levels of ligand-bound receptor were determined using a liquid scintillation counter. A thermal stability measurement (T_m_) was defined as the temperature at which 50% ligand binding was retained after a 30 min heat step at that temperature. Tm curves were analysed by GraphPad Prism v5.01 using sigmoidal dose response curve fitting.

### Half-life measurement

Samples were treated as above, except in place of the 30 minute heating step, samples were incubated at 4 °C or 10 °C for specified periods of time before separation of unbound radioligand by gel filtration. Half-life curves were analysed by GraphPad Prism v5.01 using one phase exponential decay curve fitting.

### Truncation and T4-lysozyme fusion constructs

A panel of N- and C-terminal truncation variants of CRF_1_R was designed based on secondary structure prediction (HHpred server^39^) and hydropathy plots^40^. Truncated receptors were expressed in HEK293T cells as C-terminal fusions with eGFP^41^. Receptors were solubilized in 50 mM Tris-HCl pH 8.0, 150 mM NaCl, and 2% (w/v) DM and their expression levels and stability was assayed by fluorescence-detection size exclusion chromatography (FSEC) as described previously^42^, in running buffer containing 50 mM Tris-HCl pH 8.0, 150 mM NaCl, and 0.15% (w/v) DM. In parallel, a panel of T4 lysozyme insertions into the predicted locations of ICL2 or ICL3 were analysed in a similar fashion.

### Pharmacology

For saturation binding experiments, membranes were prepared from HEK293T cells transiently expressing wild-type CRF_1_R, CRF_1_R StaR, CRF_1_R StaR with T4L fusion, or CRF_1_R-#105 as described^36^ and incubated in 50 mM Tris-HCl pH 7.5, 150 mM NaCl, 0.1% (w/v) polyethylinimine (PEI) with [^3^H]CP-376395 (0–60 nM) in the presence or absence of 30 μM cold CP-376395. Final DMSO concentration in each reaction was 5% (v/v). Membranes were incubated for 18 hours at room temperature prior to rapid filtration through 96-well GF/C UniFilter plates pre-soaked in 0.3% (w/v) PEI, followed by washing with PBS containing 0.15% (w/v) CHAPS. Plates were dried, 50 μl Ultima Gold-F scintillation fluid added per well and bound ligand measured using a Packard Microbeta counter. To obtain K_d_, data were analysed using a global fitted one-site binding hyperbola in GraphPad Prism v5. Heterologous competition analysis was conducted as above, incubating each construct with K_d_ concentrations of radioligand and a dilution series of competing ligand. To obtain K_i_, data were analysed using a globally fitted one-site fit K_i_ analysis in GraphPad Prism v5.01.

### Molecular dynamics simulations

The three dimensional coordinates of CRF_1_R in complex with the small molecule antagonist CP-376395^13^ have been downloaded from the Protein Data Bank^43^ (PDB ID: 4K5Y). The receptor has been prepared with the Protein Preparation Wizard in Maestro (Schrödinger Release 2014-2: Maestro, version 9.8, Schrödinger, LLC, New York, NY, 2014); only the protein and the ligand in chain C have been included and His155^2.50^ (Wootten numbering in superscript) has been considered to be protonated. Hydrogen atoms have been energy minimized using the OPLS2.1 force field. The conformation of missing loop residues have been predicted using Prime (Version 3.6, Schrödinger, LLC, New York, NY, 2014). The WT molecular model was created in Maestro by changing the StaR mutations to the corresponding WT residues. For these residues the side chain rotamers have been manually selected and energy minimized in Prime.

OG molecules have been prepared in Maestro and parameterized using the OPLS2.1 force field. To test the detergent behavior, a system composed by 256 OG molecules randomly located in an orthorhombic water cell including 22740 solvent molecules was created using the System Builder (Desmond Molecular Dynamics System, version 3.8, D. E. Shaw Research, New York, NY, 2014 and Maestro-Desmond Interoperability Tools, version 3.8, Schrödinger, New York, NY, 2014). The system was equilibrated using the default Desmond Relax protocol in Maestro. The final state has been subjected to 50 ns MD using DesmondGPU at 300 K/1atm in the NPT ensemble using a Nose-Hoover thermostat and a Martyna-Tobias-Klein barostat^44^ with a 2.0 ps relaxation time. Coulomb interactions were evaluated using a 9 Å short range cutoff and smooth particle mesh Ewald as long range method (Ewald tolerance = 10^−9^). During the simulation the OG molecules quickly formed a self-assembled aggregate micelle structure as expected.

A total of 224 OG molecules were manually placed using Maestro around the hydrophobic region of the CRF_1_R StaR helical bundle oriented with the polar heads toward the solvent and the hydrophobic tails toward the receptor. The system was included in an orthorhombic water cell box including 12670 water molecules and neutralized by 46 Cl^-^ and 38 Na^+^ (corresponding to 0.15 M salt concentration) using the System Builder in Maestro. After the Desmond Relax protocol in Maestro the micelle was equilibrated for 10 ns keeping the protein and ligand non-hydrogen atoms restrained using the same MD protocol described above. The WT CRF_1_R system was generated taking the obtained equilibrated OG-CRF_1_R StaR system and replacing the protein with the WT receptor model in Maestro. The total charge of the new system was neutralised removing a sodium ion and relaxed with the Desmond Relax protocol in Maestro. For both WT and StaR systems three independent 100 ns MD simulation were carried out with the same MD settings described above at a temperature of 283 K and without restrains on heavy atoms.

## Results

### StaR generation

**Figure 1:**
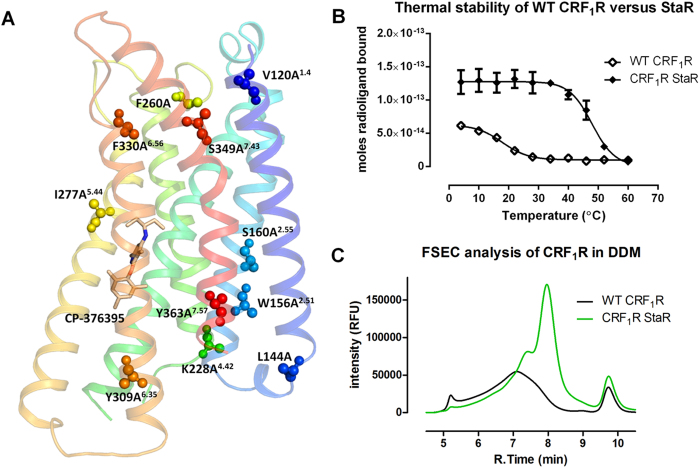
Stabilising mutations present in the CRF_1_R StaR. Panel (**A**) shows a ribbon diagram of the CRF_1_R StaR structure^50^ in complex with CP-376395 (sticks) (PDB ID: 4K5Y) coloured from TM1 in blue to TM7 in red. Positions of the stabilising mutations are highlighted in spheres and labelled with Wootten numbering in superscript. The StaR also contained the mutation S222L located in ICL2, not shown in the structure. Panel (**B**) compares the thermal stability of the CRF_1_R StaR (closed diamonds) to the wild-type receptor (open diamonds). Error bars are derived from standard deviations and calculated from duplicate temperature points within a single experiment. FSEC profiles of eGFP-tagged constructs analysed in DDM are shown in panel (**C**). Retention times of the void volume occurs at 5 min, monomeric full-length CRF_1_R at 8 min, and free eGFP at ~10 min.

Wild type CRF_1_R is highly unstable when solubilised from the lipid bilayer in detergents, precluding its use in structural studies. We have generated a conformationally stabilised CRF_1_R through mutagenesis and thermostability profiling in the presence of the inverse agonist CP-376395. Residues 114–415 of CRF_1_R were one by one mutated to alanine and transiently expressed in mammalian HEK293T cells. Where an alanine was present in the sequence to begin with, it was mutated to leucine. Detergent solubilised receptors were then analysed for thermal stability using radiolabelled [^3^H]CP-376395. A total number of 79 stabilising point mutations were identified, displaying an increase in T_m_ > 1 °C. The stabilising mutations were located throughout the receptor with the highest proportions found in TM1, TM2, TM5 and TM6. These mutations were combined to create a stabilised receptor containing 12 mutations; V120A^1.40^, L144A, W156A^2.51^, S160A^2.55^, S222L, K228A^4.42^, F260A, I277A^5.44^, Y309A^6.35^, F330A^6.56^, S349A^7.43^, Y363A^7.57^ (Wootten numbering in superscript) (Fig. 1). S222 was initially mutated to alanine, however subsequent mutation to leucine was found to be more favourable. This StaR shows a significantly enhanced stability compared to the wild type receptor, increasing the stability by 26 °C when solubilised in DDM. The StaR also shows a higher level of expression and a much improved FSEC profile (Fig. 1). While the wild type receptor largely aggregates into multimeric species, the StaR displays increased monodispersity eluting predominantly as a monomeric species. The StaR retains a shoulder to the main elution peak, however truncation of the receptor at both the N- and C-termini resolves the elution profile into a single, symmetrical peak, indicating that a proportion of instability may be driven by the N-terminal globular domain^13^.

### Stability profiling

Vapour diffusion crystallography of membrane proteins requires purification in an appropriate detergent to allow crystal contacts to form. Long-chained detergents such as DDM, while being sympathetic to protein stability, also have large micelles and can occlude the solvent exposed surface of membrane proteins. Receptor solubilised in DM was captured on Ni-NTA agarose resin and washed with a range of shorter-chained detergents to exchange the detergent associated with the receptor. Subsequent T_m_ analysis of these crudely purified samples revealed that the superior thermal stability of the StaR compared to wild type receptor was maintained across all the detergents tested (Table 1). The stability of wild type receptor is only measurable when solubilised in DDM, whereas the StaR shows an equivalent T_m_ in the significantly harsher conditions of OG (18 °C). The thermal stability of the CRF_1_R StaR was compared against the A_2A_ StaR2^36^, as a benchmark for successful crystallisation (Sup Figure 1). The CRF_1_R StaR showed an equal or higher stability than A_2A_ StaR2 in all detergents tested except OG. A correlation between detergent chain length and stability is observed in both the final CRF_1_R StaR and a preliminary StaR containing only nine of the stabilising mutations (W156A^2.51^, S222L, K228A^4.42^, F260A, I277A^5.44^, Y309A^6.35^, F330A^6.56^, S349A^7.43^, Y363A^7.57^) with the lowest stability observed with the shorter-chained detergents. This is supported by time course activity measurements of both the preliminary and final StaR (Table 2, Sup Figure 2). Detergent exchanged receptor was incubated at either 4 °C or 10 °C and the activity of the receptor was determined at various time points. These temperatures were chosen due to their applicability to current protein crystallisation techniques. The preliminary StaR displayed an activity half-life of 10 days in DM when incubated at 4 °C, which dropped to 3.5 days when the temperature was raised to 10 °C. The final StaR showed a significantly longer half-life, increasing to 22 days at 4 °C in DM, and almost 10 days when incubated at 10 °C. The half-life of the StaR reduced with shorter-chained detergents but the StaR was still 50% viable after 1.5 days at 4 °C in OTG. Taken together these data indicate that in contrast to the WT receptor, CRF_1_R StaR exhibits biochemical properties compatible with vapour diffusion crystallisation.

### Construct optimisation for crystallisation

**Figure 2:**
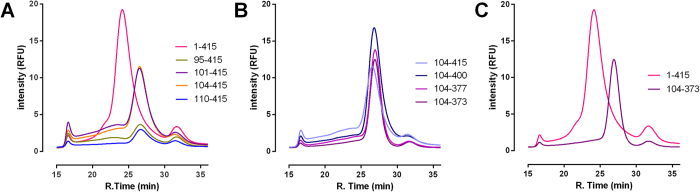
FSEC analysis of CRF_1_R StaR truncated constructs. Traces are shown representing a matrix of N-terminal truncations (panel (**A**)), C-terminal truncations based upon an N-terminal Δ103 construct (panel (**B**)), and the final truncated construct compared to the full length receptor (panel (**C**)). All constructs were analysed in the absence of ligand. Retention time of the void volume occurs at 16 min and free eGFP at ~32 min.

To aid the crystallisation of CRF_1_R the receptor was truncated at both the N- and C- termini. A matrix of truncation positions were created based upon secondary structure prediction and hydropathy plots, and screened for expression and thermal stability by FSEC. The N-terminal truncations focussing around the junction between the N-terminal globular domain and the proposed start of TM1 appeared to be quite sensitive (Fig. 2). Expression levels suffered for all N-terminal truncations, however a compromise of truncation stringency and expression was reached with NΔ103 whilst retaining a monodisperse elution profile. C-terminal truncations using this background had less of an impact on expression levels, however the FSEC elution profiles were improved. The optimal truncation position NΔ103/CΔ42 removed both the N-terminal globular domain and the predicted C-terminal helix 8. Truncation of the receptor had no observable effect on CP-376395 ligand affinity (Sup. Figure 3) but significantly improved the FSEC profile into a single, symmetrical peak.

To aid in crystallisation of the receptor in lipidic cubic phase, T4 lysozyme was inserted into ICL2 between residues T220 and D224 (construct CRF_1_R-#105^13^). The fusion caused a decrease in stability by ~7 °C, however the pharmacology of this construct remained unchanged compared to the wild type receptor (Sup. Figure 3, sup. Table 1) and generated high resolution diffracting crystals^13^.

### Buffer optimisation

**Figure 3:**
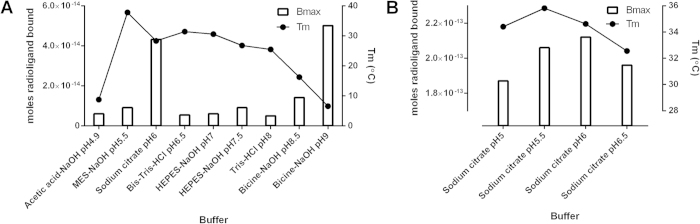
Buffer screen for thermostability. T_m_ measurements (closed circles) of CRF_1_R-#105 and radioligand B_max_ data (bars) are shown for a range of buffer conditions, panel (**A**), and a focussed sodium citrate screen, panel (**B**). Samples were analysed in DM.

The final crystallisation construct was analysed for thermal stability in a range of different buffers and additives to identify stabilising conditions for purification and crystallisation. Initial screens suggested that CRF_1_R-#105 material showed a higher stability at lower pH (5.5-7) (Fig. 3). As different buffering salts were used based upon their optimum buffering capacities, the stability of the receptor may have been influenced by the specific buffer used. Indeed, two buffers stood out; sodium citrate and Bicine pH9 gave significantly higher radioligand binding than the others tested. Fine tuning of the buffer pH revealed sodium citrate at pH5.5 to be the most stabilising. A number of additives were then screened for further improvements in stability, comprising both monovalent and divalent salts (Table 3). Divalent cations were generally destabilising, although this could be a result of the chelating properties of the divalent anion of citric acid, which at pH5.5 would form a large proportion of the ionic distribution (approx. 40%). Lithium citrate proved to be the most stabilising additive, increasing both the B_max_ and T_m_ (Table 3).

### CRF1R molecular dynamics simulation

**Figure 4:**
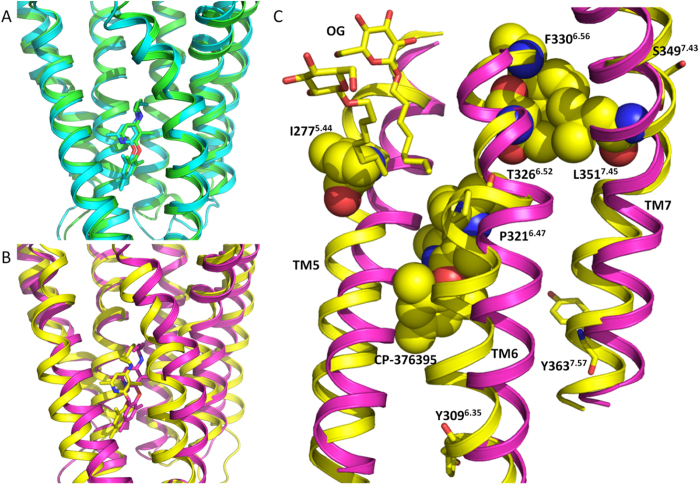
CRF_1_R WT and StaR molecular dynamics analysis. CRF_1_R structural superimposition of the starting and final conformation (after 100 ns explicit all-atom MD in an OG-water-micelle environment) for the StaR (panel (**A**)) and WT (panel (**B**)). Only the helical bundle backbone is shown as ribbon together with the ligand CP-376395. The initial conformation is coloured in green and magenta respectively for the StaR and the WT, while the final state is respectively in cyan and yellow. (panel (**C**)) Molecular destabilizing effects at the end of the CRF_1_R WT MD simulation (in yellow) of the residues I277^5.44^, Y309^6.35^, F330^6.56^ and Y363^7.57^ (all mutated to Ala in the StaR receptor). For clarity only TM5, TM6 and TM7 are shown. The backbone of the starting conformation is included in magenta as ribbon. I277^5.44^, F330^6.56^, T326^6.52^, L351^7.45^ and the ligand CP-376395 are shown as space filling, while Y309^6.35^, Y363^7.57^, P321^6.47^, S349^7.43^ and 2 OG molecules in stick representation.

To better understand the difference in thermostability between the CRF_1_R StaR and the WT receptor we analysed their conformational changes occurring after 100 ns MD at 10 °C in the harsh detergent OG (Sup. Figure 4, E). In these conditions CRF_1_R StaR is stable, whilst the WT receptor quickly unfolds (Table 1). In both systems the detergent molecules create a stable micelle around the hydrophobic regions of the TM domain. However, the helical bundle shows a difference in macroscopic behaviour between the StaR and the WT receptor (Figs 4A,B). In the first case, the crystallographic conformation is very stable, while the three independent WT simulations show the initial signs of instability and unfolding even in this relative short time frame. In this system the initial steps of the receptor unfolding are variable, but generally involve TM4, TM5, TM6 and TM7 (Table 4). The increase in the TM stability of the StaR compared to that of WT during the simulations appeared variable and not linked to the number of mutations in the helices. This supports the conclusion that the effect of the StaR mutations on the conformational rigidity is complex, and relates to the whole helical bundle, not just a single TM in isolation. Particularly remarkable was the difference in TM5, TM6 and TM7 conformational stability between WT and the StaR. During the WT MD simulations the presence of isoleucine at position 277^5.44^ (mutated to Ala in the StaR receptor) can promote the insertion of OG molecules between TM5 and TM6 close to the CP-376395 binding site (Figs 4C). In that region, TM6 shows a distorted conformation close to P321^6.47^ as a result of opposing forces acting at the extracellular and intracellular sides, in addition to the OG insertion. In the extracellular portion of the receptor we identified the hydrophobic collapse of F330^6.56^ on T326^6.52^ and L351^7.45^. The intracellular conformation of TM6 and TM7 are kept close to the helical bundle by interactions created by Y309^6.35^ and Y363^7.57^ with TM2 and TM3. Together these interactions cause a conformational strain, resulting in the bending of TM6 at position 6.47, followed by the insertion of the OG molecule. This instability is not detected in the StaR MD simulation probably as a consequence of the StaR mutations I277A^5.44^, Y309A^6.35^ and Y363A^7.57^.

The stabilising effect caused by mutations in the loops appears to be more complex and the length of the MD simulation is not long enough to allow reliable sampling of the high conformational space accessible to the highly flexible loops. However, the stabilisation could be linked to a change in loop entropy (see below).

## Discussion

The stabilisation of CRF_1_R was pivotal in the elucidation of its crystal structure^13^. The WT receptor is unstable when solubilised in detergent and like all GPCRs exists in an equilibrium between a number of different conformations. The inability to purify a homogeneous preparation of WT CRF_1_R in detergent precludes it from high resolution structural studies. The process of StaR generation not only increases the stability of the receptor and its activity in a detergent environment, but because stabilisation is driven by the interaction of the receptor with a ligand, the resulting StaR is locked into a single conformation determined by the ligand used. The CRF_1_R StaR is 26 °C more stable than the wild type receptor in DDM and is locked into an inactive conformation by the small molecule inverse agonist ligand CP-376395. The affinity of CP-376395 is slightly increased at the full length StaR however the ligand binding properties of a range of small molecule antagonist ligands appear to be unchanged. Conversely, the affinity of the StaR for the peptide agonists CRF and sauvagine is severely compromised (data not shown). This is consistent with the arrangement of the helical bundle observed in the crystal structure of the CRF_1_R StaR which clearly showed the structure of the receptor to be in the inactive conformation.

There is a correlation between the propensity of a protein to crystallise and its stability^45^,^28^. Indeed this was highlighted by the stability profiling of the final construct. Buffer and additive screening identified sodium citrate pH5.5 and lithium citrate as stabilising conditions, and interestingly the crystallisation conditions that yielded diffraction quality crystals comprised the same constituents^13^. However stability is not the only determinant of crystallisability. The final construct used in crystallisation was highly truncated, through removal of the N-terminal globular domain and predicted C-terminal helix 8, and modified by insertion of T4-lysozyme fusion into ICL2. These modifications aimed to reduce flexibility within the molecule and increases solvent exposed surface area to maximise the chances of crystallisation. Indeed, removal of the N-terminal globular domain provided a marked improvement in receptor monodispersity. This domain is a major component of agonist peptide ligand binding in CRF_1_R and its structure has been determined from refolded material expressed in *E. coli*^26^,^25^. The soluble domain of the homologue CRF_2_R has also been solved by NMR^46^,^47^ which reports a high level of dynamic flexibility within the molecule. Insertion of T4 lysozyme into ICL2 did not have a marked effect on the pharmacology of ligand binding but expression of the receptor was significantly reduced.

The mechanism by which the StaR mutations stabilise CRF_1_R in the inactive conformation is unclear. However MD simulations *a posteriori* comparing the CRF_1_R stabilised structure to a derived model of the wild-type receptor hint at possible routes of instability. The results of explicit-solvent all-atom MD analysis of CRF_1_R in a detergent micelle showed that the conformation of the helical bundle of the StaR receptor is more stable than the WT, in agreement with the experimental data. The simulations suggested that TM5 and TM6 are in particular stabilised by the StaR mutations F330A^6.56^, I277A^5.44^, Y309A^6.35^ and Y363A^7.57^. These mutations help to prevent the insertion of a detergent molecule between TM5 and TM6 close to the ligand binding site and the proline kink at position 6.47. The thermostabilising effect on the loops is harder to rationalise but it is possible that once the TM bundle has been rigidified, one way to absorb thermal energy without compromising structural integrity is to increase entropy where it can be afforded, and conceptually, loops fall into that category given that they are generally flexible. Thermophile proteins have been shown to exhibit an entropic stabilisation characteristic compared to their mesophile homologues^48^. This could be achieved through two possible mechanisms reducing the difference in entropy between the folded and unfolded state: (I) increasing the flexibility of the loop in the folded state, implying an increased conformational entropy favourable to thermodynamic stability, or (II) decreasing the entropy of the unfolded state. In the unfolded state, glycine, without a β-carbon, is the residue with the highest conformational entropy. Proline, which can adopt only a few configurations and restricts the configurations allowed for the preceding residue, has the lowest conformational entropy^49^.

Conformational stabilisation is a powerful technique that is enabling for the structural determination of unstable GPCRs. GPCRs are dynamic proteins that convey signals across the cell membrane through structural rearrangement. The introduction of 12 point mutations in CRF_1_R allowed the stabilisation of the inactive conformation of the receptor whilst leaving the ligand binding pocket unchanged. Further construct optimisation to remove flexibility combined with detergent screening and buffer optimisation allowed the successful purification and ultimately structure determination of the first Class B GPCR.

## Additional Information

**How to cite this article**: Kean, J. *et al.* Conformational thermostabilisation of corticotropin releasing factor receptor 1. *Sci. Rep.* **5**, 11954; doi: 10.1038/srep11954 (2015).

## Supplementary Material

Supplementary Information

## Figures and Tables

**Table 1:** Thermal stability analysed in a range of detergents.

Detergent	Tm preStaR(°C)	Tm StaR(°C)
DM	32	39.5
NM	25	32
NG	17	31
OG	–	18
HEGA10	25	36.5

Apparent thermal stability (Tm) values are shown for the CRF_1_R StaR and a pre-StaR construct containing only 9 of the final 12 mutations.

**Table 2:** Time course activity measurements of the CRF_1_R StaR and pre-StaR in a range of detergents.

Detergent	Half-life 4 °C (days)		Half-life 10 °C (days)	
	pre StaR	StaR	pre StaR	StaR
DM	10.2	21.9	3.6	9.6
NM	1.5	3.0	0.5	1.5
NG	0.8	2.2	0.3	1.2
OTG	0.4	1.5	0.2	0.7
C_8_E_4_	0.4	0.6	0.3	0.2
LDAO	0.3	0.8	0.2	0.4

Half-life measurements are shown in days for the CRF_1_R StaR and a pre-StaR construct containing only 9 of the final 12 mutations.

**Table 3:** Additive screen for thermal stability.

	% B_max_	ΔTm (°C)
Lithium chloride	120	−1.5
Lithium sulphate	139	1
Lithium citrate	135	2.5
Sodium sulphate	144	0.7
Sodium phosphate	98	0.9
Sodium fluoride	104	−0.7
Sodium nitrate	84	0.8
Sodium malonate	126	−1.5
Sodium tartrate	132	1.2
Sodium potassium tartrate	119	1.2
Potassium chloride	111	−0.4
Potassium phosphate	121	−0.1
Ammonium sulphate	108	0.6
Ammonium acetate	96	0
Ammonium citrate	118	0.9
Magnesium chloride	102	−7.8
Magnesium nitrate	59	−18.6
Magnesium sulphate	100	−3.7
Zinc chloride	15	−7.2
Zinc sulphate	17	−4.7
Calcium acetate	93	0.1
Nickel sulphate	58	−17.5

Radioligand binding B_max_ of construct CRF_1_R-#105 is shown as a percentage of the no additive control and the effect of each additive is represented as a change in stability compared against the no additive control.

**Table 4:**
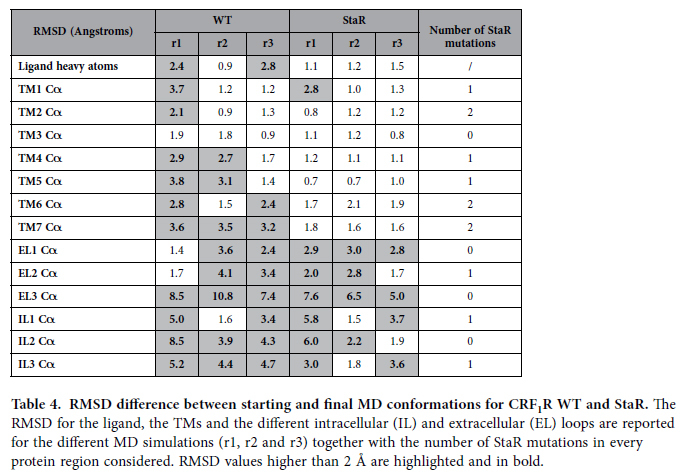
RMSD difference between starting and final MD conformations for CRF_1_R WT and StaR.
