# Supplementary material for: Conformational thermostabilisation of corticotropin releasing factor receptor 1
Source: Sci Rep. 2015 Jul 10;5:11954. doi: 10.1038/srep11954 (PMC4498186; doi:10.1038/srep11954)
Supplement: Supplementary Information [file srep11954-s1.pdf]

Conformational thermostabilisation of corticotropin releasing factor receptor 1

James Kean[1], Andrea Bortolato[1], Kaspar Hollenstein[1], Fiona H. Marshall[1] and Ali Jazayeri[1*]

[1]Heptares Therapeutics Ltd, BioPark, Broadwater Road, Welwyn Garden City, AL7 3AX, UK.

*Correspondence to: ali.jazayeri@heptares.com

Supplementary Figure 1. Thermal stability curves comparing CRF_1R StaR to $A_{2A}R$ StaR2. Tm measurements for each StaR in a range of detergents are displayed in the inserted table. Error bars are derived from standard deviations and calculated from duplicate temperature points within a single experiment.

Supplementary Figure 2. Time course activity curves for the StaR and pre-StaR. Example curves are shown for samples incubated at 10°C in DM, NM, NG and OTG.

Supplementary Figure 3. Saturation binding analysis of [^{3}H]CP-376395. Total binding curves (closed circles) are shown representing wild type CRF_1R, the full length StaR, full length StaR with T4 lysosyme insertion, and the final truncated crystallisation construct #105, compared against untransfected controls (open circles). Specific binding curves are shown as dashed lines. Calculated K_d (nM) are 7.5(2.4), 1.5(0.1), 6.7(2.5), and 5.0(0.8) respectively with standard deviation in parentheses.

Supplementary Figure 4. Molecular arrangement difference between membrane lipids and detergents. Schematic molecular orientation around a protein (blue rectangle) of a lipid bilayer (panel **A**) and a detergent micelle (panel **C**) (shown as circle representing the polar group connected to a line representing the lipophilic tail). Molecular representation of CRF_1R (green surface) embedded in a DMPC lipid membrane (in yellow) (panel **B**) or in an OG (in yellow) micelle (panel **D**). Water molecules are shown as small red spheres and hydrogen atoms have been hidden for clarity. (panel **E**) The molecular structures of a DMPC lipid (top) and different detergents (DM, HEGA10, NM, NG, OG respectively from top to bottom) are shown.

Supplementary Table 1. Heterologous competition analysis of CRF_1R antagonist ligands against [^3H]CP-376395. Calculated K_i (nM) are shown for wild type CRF_1R, the full length StaR, and the final truncated crystallisation construct #105, with standard deviation in parentheses.

Ligand	WT	StaR	Construct#105
Antalarmin	4.2(1.4)	5.1(1.7)	8.7(5.0)
NBI 35965	2.0(0.2)	2.8(0.7)	9.5(0.4)
SN 003	10.4(4.6)	8.7(1.7)	28.6(31.0)

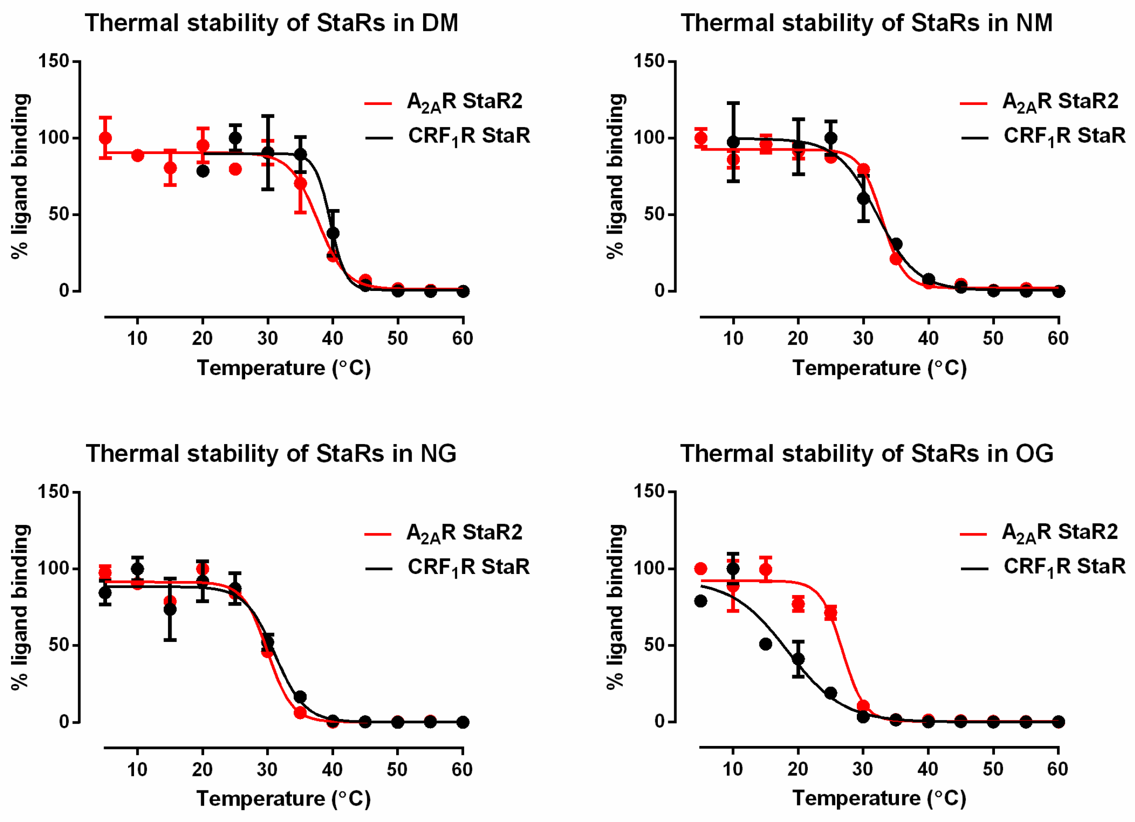

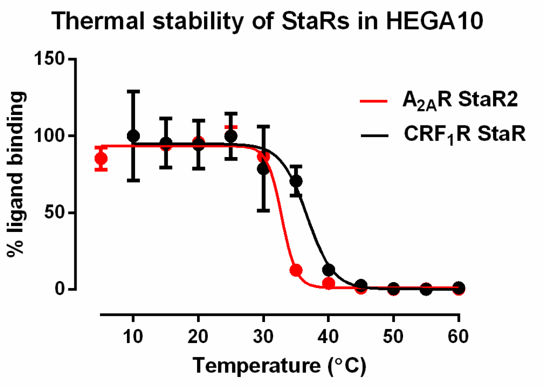

Detergent	Tm CRF1R StaR (°C)	Tm A_{2A}R StaR2 (°C)
DM	39.5	37.5
NM	32	32.5
NG	31	30
OG	18	26.5
HEGA10	36.5	32.5

Supplementary Figure 1

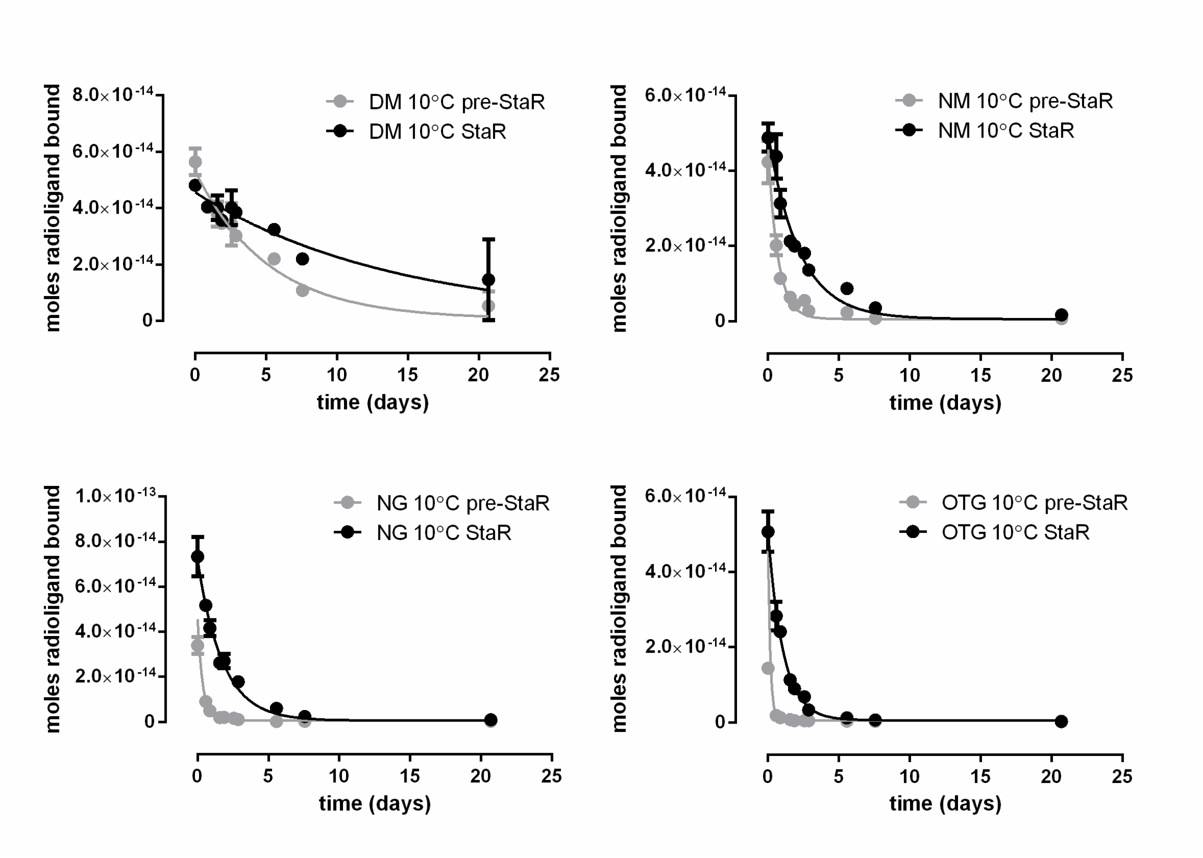

Supplementary Figure 2

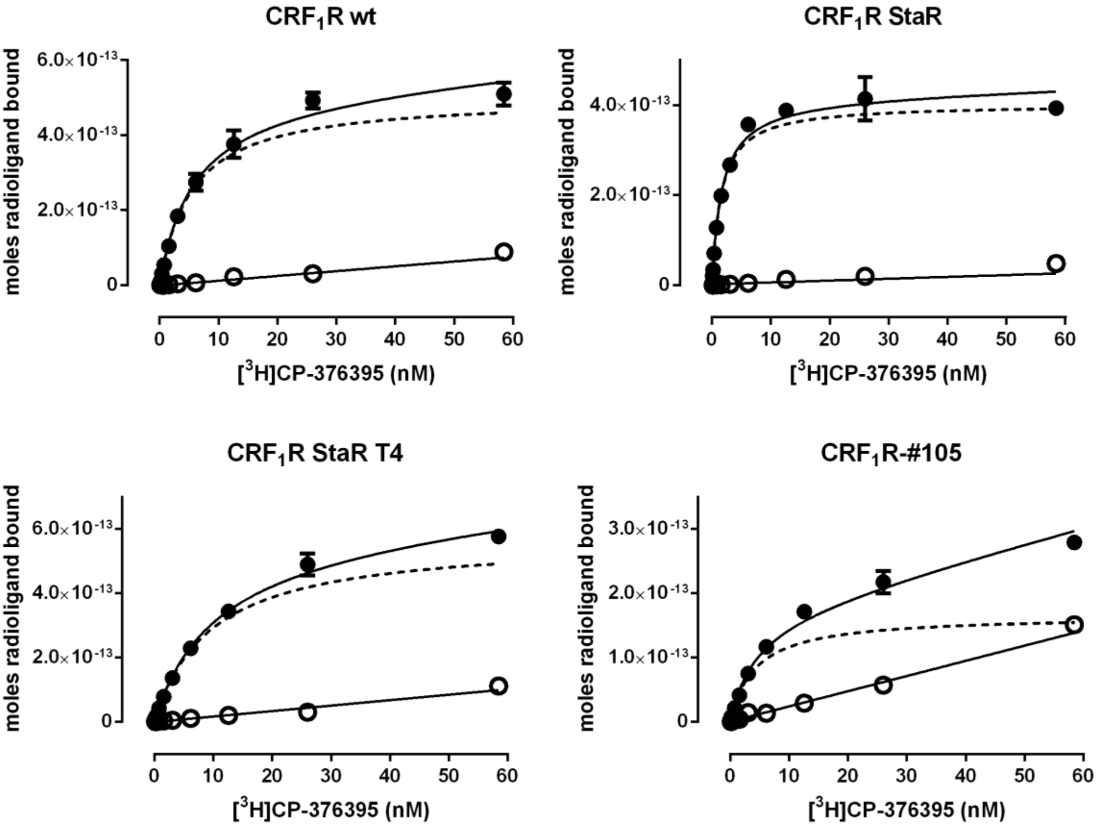

Supplementary Figure 3

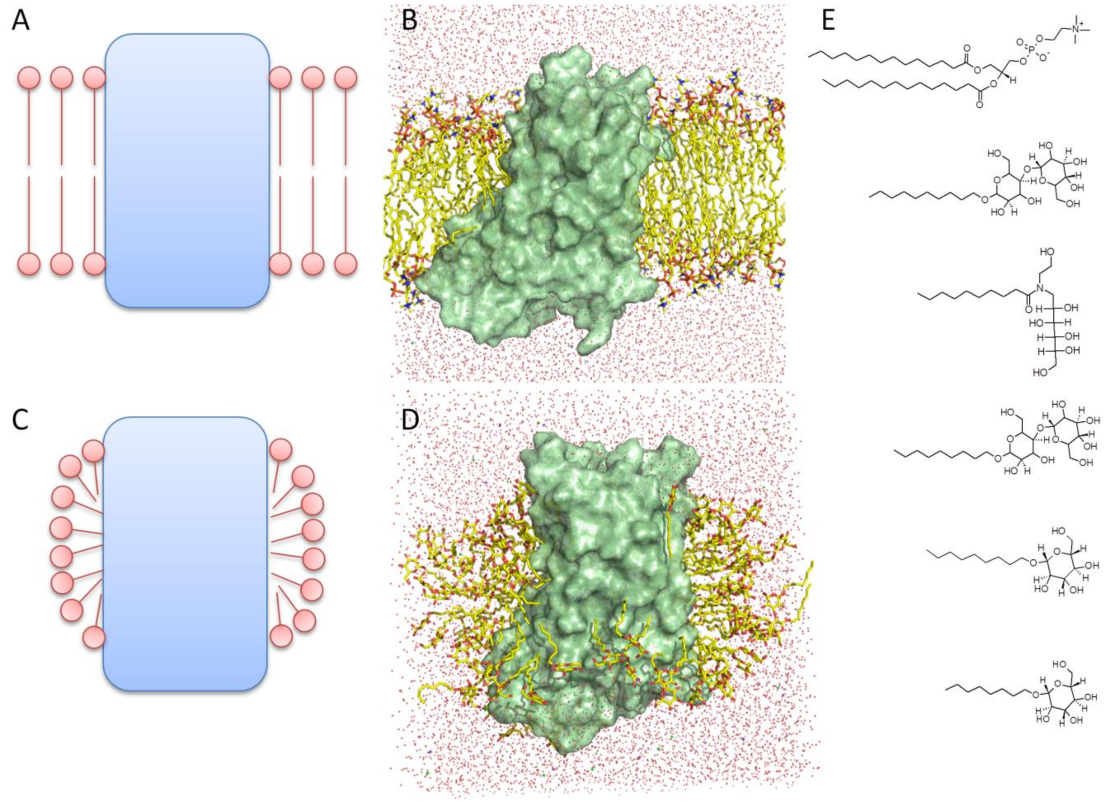

Supplementary Figure 4
